# Recommendation of rotavirus vaccination and herd effect: a budget impact analysis based on German health insurance data

**DOI:** 10.1007/s10198-014-0624-2

**Published:** 2014-08-19

**Authors:** Alexander Karmann, Andrea Jurack, Daniel Lukas

**Affiliations:** 1 Fakultät Wirtschaftswissenschaften, Gesundheitsökonomisches Zentrum, TU Dresden, 01062 Dresden, Germany; 2 Lehrstuhl für Allokationstheorie, Fakultät Wirtschaftswissenschaften, TU Dresden, 01062 Dresden, Germany; 3 Lehrstuhl für Wirtschaftsforschung und Wirtschaftspolitik, Fakultät Wirtschaftswissenschaften, TU Dresden, 01062 Dresden, Germany

**Keywords:** Rotavirus, Vaccination, Germany, Herd effect, Budget impact analysis, H51, I13, I18

## Abstract

The objective of this study was to assess the budget impact and health effects of introducing rotavirus (RV) vaccination in Saxony, Germany, from a health insurance perspective. Special emphasis is given to the herd effect. We analyzed direct medical and non-medical costs of RV infection for Social Health Insurance between 2007 and 2010 based on 360,000 routine data observations from the AOK PLUS for children below 5 years of age. We compared the actual annual number of RV cases (vaccination scenario) with the number derived from 2005 (no vaccination, base case scenario). The vaccination coverage rate has increased from 5 % to 61 % between 2007 and 2010. The number of RV cases decreased by 21 % from 32,274 in 2007 to 25,614 in 2010. Based on vaccination coverage, the total cost savings per 1,000 children due to RV vaccination was estimated to be 39,686 Euros. The overall share of outpatient costs was 60 %. Mean gross cost savings were expected to be 304 Euros per avoided case. The net cost savings were expected to be 19 Euros per avoided case. About 59 % of total savings was due to herd protection resulting from increasing vaccine rates. The herd effect per avoided case increased with increasing vaccine coverage. Incidence of RV cases, vaccination costs and days absent from work were sensitive parameters. This retrospective analysis showed that the increase in RV vaccination coverage in Saxony has been budget neutral if not cost saving for sick funds.

## Introduction

In Germany, rotavirus (RV) was the third most commonly reported cause of acute gastroenteritis (GE) during 2005 and 2010 and was the leading cause of acute GE in the last decade in children below 5 years of age [1]. In Germany, laboratory-confirmed RVGE has been a notifiable disease since 2001 [1, 2]. In industrialized countries RVGE does not lead to death; however, the burden of RVGE disease remains considerable resulting in frequent outpatient visits and hospitalization. Consequently, the medical and economic burden associated with RVGE in these countries is high [3–7]. As with other developed nations, RVGE disease burden constitutes a significant public health problem in Germany [1, 2, 6, 7]. Of the total number of RVGE cases reported in Germany in children below 5 years of age, 57 % were hospitalized [2, 8].

Two orally administered RV vaccines, which have demonstrated to have good efficacy and safety in global clinical trials, have been licensed in most countries: Rotarix^®^ (GlaxoSmithKline Vaccines, Wavre, Belgium) and RotaTeq^®^ (Merck, Whitehouse Station, NJ) [9–12]. These vaccines were licensed by the European Medicines Agency (EMA) for use in Europe in 2006 [5, 13]. Rotarix^®^ and RotaTeq^®^ are available in a two- and three-dose schedule, respectively. The vaccine doses are prescribed as follows: the first dose is given to children from the age of 6 weeks until 24 weeks for Rotarix^®^ and 32 weeks of age for RotaTeq^®^. In 2009, the World Health Organization (WHO) recommended that vaccination against RV should be implemented through universal routine immunization of infants. Additionally, establishment of surveillance systems to monitor vaccine impact where RV vaccines are available was also included in the WHO recommendation [14, 15].

Since the availability of RV vaccines in Germany, RV vaccination coverage across the federal states in Germany has increased steadily and reached 25 % in 2010 [3]. In Saxony, 61 % of all children were vaccinated against RV in the same year [16]. Five federal states of Germany, including Saxony, have been releasing local vaccine recommendations for the prevention of RVGE disease in young children. A recommendation for routine rotavirus of the German Standing Committee on Vaccination (STIKO) has been published in 2013 [17]. At the time this analyses was initiated health insurances companies were therefore not obliged to reimburse vaccination costs [8].

To assess the economic effects of RV vaccination, cost-effectiveness analyses based on Markov cohort models are typically used (see e.g., Standaert et al. [18] for RV hospitalizations in Belgium [18]). Despite the presence of an adequate infrastructure and a robust surveillance system in place in Germany [1, 8], there is limited evidence on the cost benefit of implementation of RV vaccination [16], especially from the payer perspective. The aim of this analysis was therefore to quantify the financial effects of the implementation of universal RV vaccination in Saxony based on observed RV cases, with emphasis on the direct effects of vaccination and protection induced from herd effects as a positive externality of vaccination coverage.

## Methods

### Model structure

We performed a retrospective budget impact analysis (BIA) on the implementation of RV vaccination in Saxony from the Statutory Health Insurance (SHI) perspective. We evaluated the benefits of implementation of RV vaccination (two-dose schedule) in children below 5 years of age including protection conferred as a result of herd effect. The BIA was quantified by a comparison of costs between base case and vaccine scenario. Total cost savings (in Euros) per 1,000 children and cost savings (in Euros) per avoided case were estimated. Cost savings were also estimated for both inpatient and outpatient groups and quantified separately due to herd and direct effects of vaccination. We conducted the analysis in MS Excel via a three step approach:

1. Calculation of inpatient and outpatient cases and associated costs based on AOK PLUS data.
2. Extrapolation of RV cases in base case scenario.
3. Comparison of vaccination scenario and base case scenario on in- and out-patient cases as well as associated costs.
4. Deduction and estimation of observed herd effect.

### Input data

#### Epidemiological data

The RV cases selected for the present analysis are based on data from a statutory German health insurer—AOK PLUS [19]. About 55 % of Saxon children from the 1st until the 5th year of life are insured by AOK PLUS. We analyzed a relatively large data set for the vaccination scenario that was based on 360,000 observations including number of cases and costs per case of RVGE for the years 2007–2010 for both inpatient and outpatient settings.

In Germany, laboratory-confirmed RV infections are notifiable. However, mild and moderate infections are often managed at home without consulting a physician. In case of treatment physicians usually do not perform testing [20]. As a result, the number of notified cases is much lower than annual incidence rates reported in the literature [4, 21–24]. Hence, reported RV infections are likely to underestimate the true number of cases [25]. Therefore our AOK PLUS observations included the following diagnoses according to the International Classification of Diseases, Tenth Revision (ICD-10) codes 2011: A08.0: Rotaviral enteritis, A08.3: Other viral enteritis, A08.4: Viral intestinal infection, unspecified and A09: Infectious gastroenteritis and colitis, unspecified. A case of RVGE for the purpose of this analysis was defined as one or more treatments of an insured person by the same physician within a quarter of a year (i.e., even if an insured person received more than one treatment within the same quarter of a year, this will be handled as one case only).

With the given data we could not identify whether a specific child received outpatient or inpatient treatment within the same quarter of the year. Consequently, our data does not represent the actual number of patients but rather the number of cases registered in each of the two treatment groups, i.e., outpatient and inpatient group. It should also be noted that the inpatient group includes the patient contact to emergency. Statistical methods to differentiate cases are not available, but we refer to Giaquinto et al. [2], stating that contact with emergency is always associated with hospitalization.

Since the time period in the AOK PLUS data set was limited to 2007–2010, we also used data from the Robert-Koch-Institut (RKI) [26] for Saxony to calculate the percentage increase in RVGE numbers for the years 2003–2007. It is important to note that cases identified from the RKI include only the diagnosis ICD-10 code A08.0: Rotaviral enteritis. Sources for the input data are given in Table 1.

**Table 1:** Input data

Input factor	Source/assumption
Population	Statistical Office of Saxony
Vaccination coverage	[3, 16]
RV cases, vaccine scenario (2007–2010)	AOK PLUS data set [19]
RV cases, base case scenario (2007–2010)	AOK PLUS cases from 2007 counted back to 2005, then calculation of hypothetical cases (2007–2010) using average RKI growth rates (2001–2007)
Percentage annual increase in RV cases (2001–2007)	RKI data set [26]
Direct medical costs	AOK PLUS [19]
Direct non-medical costs (sickness-benefits)	
Rate of employment of mothers	Statistical Office of Saxony [36]
Wage	Statistical Office of Saxony [27]
Days of absence from work	Giaquinto et al. [2]
Costs of vaccination	LAUER-TAXE^®^ [29]

* RV* Rotavirus,* RKI* Robert-Koch-Institut

#### Costs

**Fig. 1:**
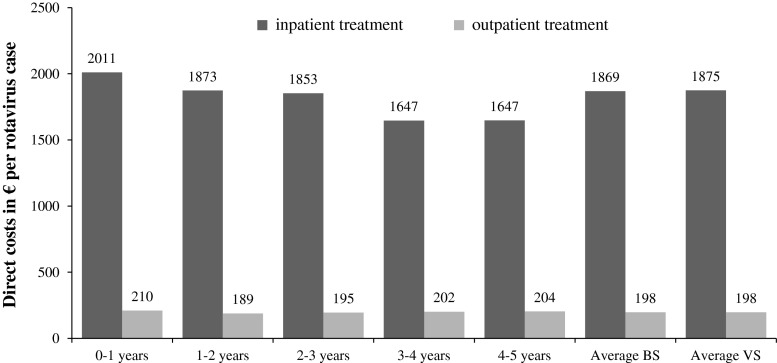
Costs per rotavirus (RV) case from a statutory health insurance (SHI) perspective in 2010

Our BIA was limited to the evaluation of costs from the SHI payer perspective. The different types of costs were calculated using data from the Federal Statistical Office, the Statistical Office of Saxony and AOK PLUS. Moreover, we used cost data from the REVEAL study [2], which analyzed the costs and burden of RV infection in another East German Federal State (Mecklenburg-Vorpommern in 2004). Figure 1 shows the total costs per case from the SHI perspective, including direct medical and non-medical costs as well as the costs for vaccination. As expected, inpatient cases are more expensive than outpatient ones. The cost structure to determine total costs per case are explained below.

##### Direct medical costs

The data set of AOK PLUS allowed for the analysis of average direct medical costs per age group, and for inpatient and outpatient treatment groups. The included cost categories, for example medication and physician visits, could not be specified any further because the data set contained only the total medical costs. From the age-group-specific costs we get average costs per case by weighting according to age distribution (Table 2).

**Table 2:** Economic burden associated with RV infection (Euros per case) and vaccination costs

Year	2007		2008		2009		2010	
Treatment group	Inpatient	Outpatient	Inpatient	Outpatient	Inpatient	Outpatient	Inpatient	Outpatient
Age group								
Direct medical costs								
0–1 years	1,737	85	1,758	64	1,813	73	1,849	76
1–2 years	1,429	66	1,593	49	1,623	53	1,712	56
2–3 years	1,409	55	1,555	45	1,621	50	1,680	52
3–4 years	1,304	48	1,468	42	1,471	48	1,463	50
4–5 years	1,294	46	1,409	44	1,441	48	1,461	49
Average BS	1,508	60	1,614	48	1,652	53	1,700	55
Average VS	1,517	61	1,618	48	1,656	53	1,707	55
Direct non-medical costs (sickness benefits, kinderkrankengeld)								
0–1 years	153	126	156	129	159	132	162	134
1–2 years	152	126	155	128	158	131	161	133
2–3 years	163	135	166	138	170	141	173	143
3–4 years	173	144	177	146	180	149	184	152
4–5 years	175	145	179	148	183	151	186	154
Costs of vaccination	124		124		124		124	

##### Direct non-medical costs (sickness benefit)

We assumed that the professional working mother stayed at home during the time of inpatient or outpatient treatment of her child. In Germany, health insurers have to pay sickness benefits to the parent during absence from work. The proportions of mothers in Saxony with children in the relevant age groups who are working professionals are shown in Table 3. The average number of days of leave of the parent from work due to RV infection of the child were assumed as follows: outpatient cases 5.3 days and inpatient cases 6.4 days. These data refer to survey results of the REVEAL study and are limited to the urban population of Rostock between 2004 and 2005 [2]. To determine the sickness benefits, we used the average gross wage per female employee (20–45 years) in Saxony in 2010 of 102.87 (full-time) and 71.13 (part-time) Euro per day in 2010 (Statistical Office of Saxony [27]). As the amount of sickness benefits depends on the net wage, we calculated the net wage for Saxony as two-thirds of the average gross wage. This value was deflated for the years 2007 to 2009 at an average rate of 2 % corresponding to economic development [28]. Assuming a sickness benefit of 75 %, the costs were calculated as follows:

$${\text{Sickness}}\,{\text{benefits}}\,_{{{\text{year,}}\, {\text{age}}}}\,\,{ = 0} . 7 5 \times {\text{net}}\,{\text{wage}}\,_{{{\text{year,}}\,{\text{age}}}} \times {\text{days}}\,{\text{absent}}\,{\text{from}}\,{\text{work }} \times {\text{rate }}\,{\text{of}}\,{\text{employment}}\,{\text{of}}\,{\text{mothers}}\,_{\text{age}}$$

**Table 3:** Percentage of mothers in Saxony who are working professionals [34]

Age of the child (years)	Employment rate of mothers (%)	Full-time (%)	Part-time (%)
0–1	52.7	77	23
1–2	54.5	64	36
2–3	59.6	60	40
3–4	31.5	59	41
4–5	65.4	58	42

##### Costs of vaccination

The total vaccination cost of 124 Euro included—for a two-dose schedule—the cost of vaccine of 58.53 Euros per dose (taken from a pack with one dose, [29]) and a medical fee of 6.50 Euros [30].

### Vaccine scenario

The vaccine scenario represents the actual number of RV cases reported subsequent to the implementation of RV vaccination in Saxony in 2006 which is given by the AOK PLUS data set for the years 2007 to 2010. It was observed that both the numbers of inpatient and outpatient RV cases decreased significantly post-introduction of RV vaccines in 2006. Additionally, we calculated the incidence rate per age group and the probabilities of the RV case seeking outpatient or inpatient care (Table 4). Table 4 presents the vaccination coverage for the years 2007–2010 in Saxony. Given the number of insured children, we estimated the number of unprotected children. We assumed for the present analysis that immunization is effective for 2 years. We excluded any vaccine waning effect as previous studies have demonstrated its absence in real life conditions as well as a high vaccine efficacy in clinical trials of nearly 100 % [9–11, 18]. Given the distribution of vaccinated children per year, we assume that 80 % of the children are protected during the first year of life. This is based on the fact that the children are not vaccinated immediately at the time of birth, but on an average 2 and 4 months of age. Therefore, we estimated 100 % protection for children of ages between 1 and 2 years and 20 % protection for children of ages between 2 and 3 years. We estimated the incidence rates as ratio of inpatient or outpatient RV cases and unprotected children (Table 4).

**Table 4:** Vaccine scenario: AOK PLUS data set for Saxony, 2007–2010

Insured children	Number of RVGE cases			
Age group/year (*n*)	2007	2008	2009	2010
0–1 years	19,094	19,044	18,093	17,970
1–2 years	18,759	19,094	19,044	18,093
2–3 years	18,240	18,759	19,094	19,044
3–4 years	17,776	18,240	18,759	19,094
4–5 years	16,484	17,776	18,240	18,759
Overall	90,353	92,913	93,230	92,960

Vaccination coverage (%)	2007	2008	2009	2010
	5	35	52	61

Unprotected children (*n*)	2007	2008	2009	2010
0–1 years	18,330	13,712	10,566	9,201
1–2 years	18,759	18,139	12,379	8,685
2–3 years	18,240	18,759	18,903	17,711
3–4 years	17,776	18,240	18,759	19,094
4–5 years	16,484	17,776	18,240	18,759
Overall	89,589	86,626	78,847	73,449

Vaccinated children (*n*)	2007	2008	2009	2010
	955	6,665	9,408	10,962

RV cases (A08.0, A08.3, A08.4, A09) (calculated incidence rates)									
Year		2007		2008		2009		2010	
Age/treatment group		Inpatient	Outpatient	Inpatient	Outpatient	Inpatient	Outpatient	Inpatient	Outpatient
0–1 years		858 (0.05)	4,767 (0.26)	782 (0.06)	4,395 (0.32)	582 (0.06)	3,548 (0.34)	578 (0.06)	3,078 (0.33)
1–2 years		744 (0.04)	9,197 (0.49)	749 (0.04)	9,267 (0.51)	622 (0.05)	8,692 (0.70)	591 (0.07)	6,624 (0.76)
2–3 years		380 (0.02)	7,177 (0.39)	399 (0.02)	7,015 (0.37)	315 (0.02)	6,477 (0.34)	314 (0.02)	5,778 (0.33)
3–4 years		229 (0.01)	5,214 (0.29)	221 (0.01)	5,664 (0.31)	183 (0.01)	4,956 (0.26)	186 (0.01)	4,793 (0.25)
4–5 years		147 (0.01)	3,560 (0.22)	159 (0.01)	4,215 (0.24)	122 (0.01)	3,489 (0.19)	125 (0.01)	3,547 (0.19)
Overall		2,358 (0.03)	29,916 (0.33)	2,310 (0.03)	30,556 (0.35)	1,824 (0.02)	27,162 (0.34)	1,794 (0.02)	23,820 (0.32)

* RVGE* Rotavirus gastroenteritis

### Base case scenario

To calculate the net effect of the vaccination program in Saxony we developed a hypothetical data set. The base case scenario considered hypothetical cases that would have occurred between 2007 and 2010 without a vaccination program. For this, we used data from RKI (Table 5) for Saxony, since the data of AOK PLUS are available only from 2007 until 2010. In detail, the base case scenario was built on the assumption that the growth rates of RKI cases are identical with that of AOK PLUS. According to these growth rates, no up- or down-ward trend can be identified for the period prior to the introduction of the vaccine (Table 5). Thus the incidence rates of RV cases were probably not influenced by external factors. The following steps were implemented in the construction of the base case scenario for each age group (Table 6):

**Table 5:** RKI data set: RV cases (A08.0) in Saxony

Number of cases								
Age group/year	2001	2002	2003	2004	2005	2006	2007	2008
0–1 years	1,051	1,682	1,877	1,383	1,941	2,096	1,794	1,485
1–2 years	2,085	1,989	1,924	1,445	2,352	2,508	2,172	2,256
2–3 years	1,183	1,065	968	643	1,064	1,262	1,072	1,278
3–4 years	632	639	530	346	594	615	541	723
4–5 years	391	383	260	216	424	374	318	443
Overall	5,342	5,758	5,559	4,033	6,375	6,855	5,897	6,185

**Table 6:** Construction of the base case scenario: RV cases in Saxony (RKI) or in AOK PLUS data set

Step 1: moving average = adjusted RKI cases per year	2005	2006	2007
	2001–2005	2002–2006	2003–2007
0–1 years	1,587	1,796	1,818
1–2 years	1,959	2,044	2,080
2–3 years	985	1,000	1,002
3–4 years	548	545	525
4–5 years	335	331	318
Overall	5,413	5,716	5,744

Step 2: % annual increase in RV cases			
0–1 years	+0.13	+0.00	
1–2 years	+0.04	+0.01	
2–3 years	+0.00	+0.00	
3–4 years	−0.02	−0.05	
4–5 years	−0.03	−0.06	
Average	+0.04	+0.00	

Step 3: counted-back AOK PLUS cases	2005	2006	2007
0–1 years	4,688 (in. = 703, out. = 3,985)	5,281	5,625
1–2 years	9,139 (in. = 640, out. = 8,500)	9,392	9,941
2–3 years	7,313 (in. = 366, out. = 6,947)	7,554	7,557
3–4 years	5,756 (in. = 230, out. = 5,525)	5,690	5,443
4–5 years	4,299 (in. = 172, out. = 4,127)	4,142	3,707
Overall	30,474	31,995	32,274

Step 4: incidence rates	Inpatient	Outpatient	
0–1 years	0.04	0.22	
1–2 years	0.04	0.47	
2–3 years	0.02	0.39	
3–4 years	0.01	0.32	
4–5 years	0.01	0.24	
Average	0.02	0.33

- To account for the seasonal fluctuation of RV cases within the RKI data set we calculated a moving average with a timespan of 5 years to derive adjusted case values for the years 2005, 2006 and 2007.
- We computed the growth rates of RV cases for the years 2006 and 2007.
- The actual numbers of RV cases from AOK PLUS from 2007 were counted back to 2005 by using the previously calculated percentage increase of the RKI cases. We assumed a constant distribution of inpatient and outpatient cases (as observed in the AOK PLUS data set for 2007–2010), following which we estimated the number of cases in both the groups—inpatient and outpatient for the year 2005.
- The simulated numbers of RV cases were considered to infer the incidence rates of RV cases for Saxony in 2005 for the inpatient and outpatient groups based on the total number of insured children per age group. We assume those to be constant and therefore also valid for 2007 to 2010.

The base year 2005 was chosen because RV vaccines were not licensed until 2006. Using the number of insured children by AOK PLUS and the calculated incidence rates, the hypothetical number of cases in the base case scenario for the years 2007 to 2010 were determined (Table 7).

**Table 7:** Base case scenario: derived hypothetical RV cases (no vaccine administered) in Saxony, 2007–2010

Year	2007		2008		2009		2010	
Age/treatment group	Inpatient	Outpatient	Inpatient	Outpatient	Inpatient	Outpatient	Inpatient	Outpatient
0–1 years	1,329	7,532	1,351	7,656	1,337	7,576	1,331	7,544
1–2 years	1,140	15,145	1,187	15,772	1,208	16,050	1,203	15,982
2–3 years	673	12,792	672	12,761	700	13,293	697	13,237
3–4 years	438	10,515	429	10,294	429	10,301	427	10,257
4–5 years	315	7,569	326	7,819	319	7,662	318	7,629
Overall	3,962	53,911	3,974	54,558	3,946	54,990	3,888	54,450

### Analyses

#### Base case versus vaccine scenario

The overall effect (RV cases and cost savings) of RV vaccination, i.e., the difference between base case scenario and vaccine scenario was reported for all children below 5 years of age. These effects were reported for both inpatient and outpatient settings as well as for children segregated into five age groups (0–1, 1–2, 2–3, 3–4 and 4–5 years).

#### Calculation of vaccination effectiveness

The total effect of RV vaccination was calculated as:

$${\text{Total}}\,{\text{effect}}\,{ = }\,{\text{RV}}\,{\text{cases (BS)}}\, - \,{\text{RV}}\,{\text{cases}}\, ( {\text{VS).}}$$

#### Herd effect and direct effect

The total effect due to vaccination can be seen as a result of vaccine direct effects and due to herd protection. The direct effect represents the expected direct cost savings for the insurance. In general, the herd effect describes the reduction in infection probability of unvaccinated individuals as a result of others in the same society being vaccinated [31]. Following that definition, we calculated the herd effect as the difference between expected RV cases in the base case scenario and actual RV cases in the vaccine scenario. It was determined as follows:

$${\text{Herd}}\,{\text{effect}}\,{ = }\,{\text{rotavirus}}\,{\text{incidence }}\, ( {\text{BS)}}\,{{ \times }}\,{\text{unprotected}}\,{\text{children}}-{\text{actual}}\,{\text{rotavirus }}\,{\text{cases }}\, ( {\text{VS)}}$$

We implemented the following steps to quantify the importance of herd effect for each age group:

- Calculation of RV incidence (BS) × unprotected people (VS) as the hypothetical number of RV cases if the incidence rate were unaffected by vaccination.
- Calculation of RV incidence (BS) × unprotected people (VS)—actual RV cases (VS) as difference between the previously calculated hypothetical cases and the actual cases taken from the VS.

If actual RV cases exceeded the number of expected RV cases, herd effect was set to zero. A positive difference between the hypothetical and actual number of RV cases implied a decrease in incidence for the VS and confirmed immunization resulting from herd protection.

The *direct effect* results from

$${\text{Direct}}\,{\text{effect}} = {\text{Total}}\,{\text{effect}} - {\text{Herd}}\,{\text{effect}}$$

which is the difference between the number of avoided cases and the herd effect. If we derived a positive total effect for the age groups 3–4 and 4–5 years, the total effect was equal to the herd effect because *direct* protection through vaccination is not feasible for these groups. Hence, the direct effect is zero.

#### Sensitivity analyses

To account for the uncertainty in our assumptions or to validate the robustness of our assumptions, we conducted a univariate sensitivity analysis by varying the sensitive parameters until the net cost savings faded out. The hypothetical cases calculated within the base case scenario are based on several assumptions. We assumed constant incidence rates for the period 2007–2010 which were estimated based on incidence rates of 2005 and which resulted from a data set including only part of diagnoses relevant for RV disease. Therefore, “data” variations in the outpatient and inpatient incidence rate of ±0.5 and ±0.06 % were analyzed. Moreover, we analyzed a variation of “days of absence from work” of ±1 and ±4 days as well as a decrease in vaccination costs of 15 %.

## Results

### Base case scenario versus vaccine scenario

**Fig. 2:**
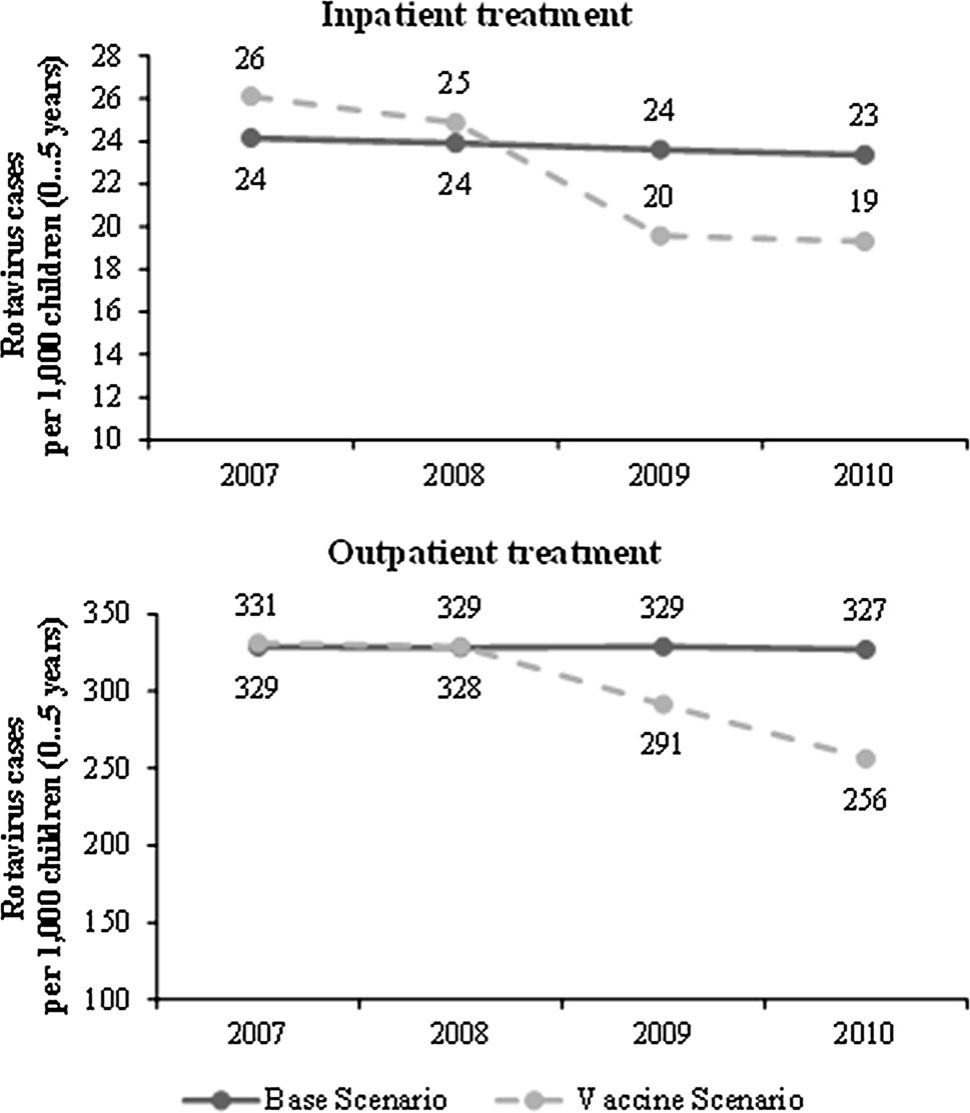
Incidence of inpatient and outpatient RV cases per 1,000 children

Table 8 shows the number of RV cases avoided for all children stratified by age group and year. As observed for the years 2007 and 2008 for some age groups, the estimated numbers of RV cases avoided are negative. As there is no plausible explanation for negative signs from a medical perspective, increasing awareness and changing communication about RV cases by medical professionals might have caused higher numbers of RV cases registered in the time period following the implementation of RV vaccination in Saxony. Indeed, for 2009, this effect no longer exists, which can be explained by increasing effectiveness of the vaccination program corresponding with a stronger decrease of cases within the vaccine scenario. Figure 2 shows the time trend curve of inpatient and outpatient RV cases for both the vaccine and the base case scenario without differentiation of age groups. The figure shows the actual data of both scenarios without the previously explained correction of numbers. Both the numbers of inpatient as well as the outpatient RV cases decreased significantly in the vaccine scenario while these numbers remained fairly constant in the base case scenario.

**Table 8:** RV cases averted per 1,000 children

Year	Category	Avoided cases = total effect		Due to herd effect		Due to direct effect
	Age group	Inpatient	Outpatient	Inpatient	Outpatient	
2007	0–1 years	0 (−5.7)	0 (−27.2)	–	–	–
	1–2 years	0 (−4.6)	0 (−24.5)	–	–	–
	2–3 years	0 (−0.2)	0 (−0.9)	–	–	–
	3–4 years	0.3	24.1	0.3	24.1	0
	4–5 years	0.9	20.7	0.9	20.7	0
2008	0–1 years	0 (−1.8)	0 (−8.3)	–	–	–
	1–2 years	0 (−4.2)	0 (−19.5)	–	–	–
	2–3 years	0 (−0.6)	18.7	0	18.7	0
	3–4 years	1.1	6.9	1.1	6.9	0
	4–5 years	0.9	0 (−0.4)	0.9	0	0
2009	0–1 years	7.1	26.4	0	0	33.5
	1–2 years	2.4	9.4	0	0	11.8
	2–3 years	4.2	53.4	4.0	49.4	4.1
	3–4 years	3.5	53.2	3.5	53.2	0
	4–5 years	3.2	45.4	3.2	45.4	0
2010	0–1 years	7.1	51.2	0	0	58.3
	1–2 years	2.4	99.7	0	0	102.1
	2–3 years	4.2	89.2	2.7	61.7	29.0
	3–4 years	3.5	66.4	3.5	66.4	0
	4–5 years	3.2	47.6	3.2	47.6	0
2007–2010		44	612.3	23.3	394.1	238.8

**Fig. 3:**
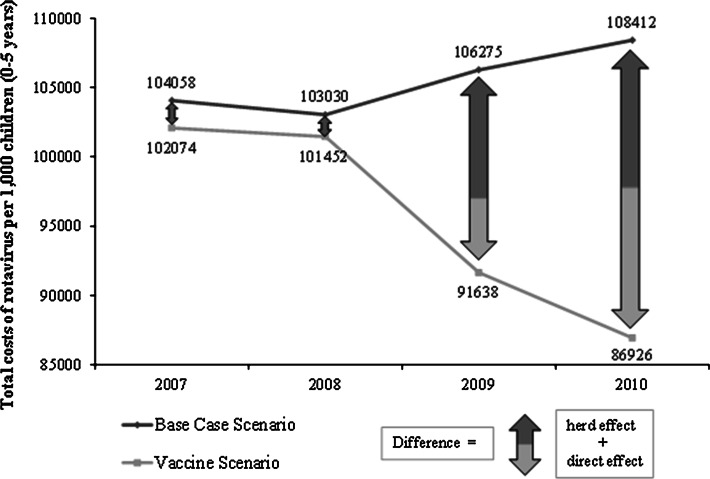
Illustration of the herd effect

Given that the total number of vaccinated children is 27,990, which was estimated as the product of vaccination coverage rate and total population of children within the AOK PLUS data set, the sum of avoided cases was estimated to be 12,143. Total costs per 1,000 children from the SHI perspective are shown in Table 9. In the base case scenario, the costs remained almost constant while in the vaccine scenario they decreased from 102,074 to 86,926 Euros per 1,000 children (Fig. 3). This decrease resulted mainly from the decline in the number of RV cases. Given that the vaccination coverage rate has increased from 5 % to 61 % in the observation period, we obtained the following estimates: the expected total cost savings attributable to implementing RV vaccination in Saxony during 2007 and 2010 was 2,477 Euros per 1,000 children from the SHI perspective. These savings already account for vaccination costs. The proportions of outpatient and inpatient costs of the total costs were about 60 % and 40 %, respectively. The overall mean gross cost savings per avoided case due to vaccination during the observation period was estimated as 292 Euros per avoided case (Table 9). The ratio between numbers of vaccinated children and RV cases avoided due to vaccination were used to estimate the effective costs per vaccination, which was 304 Euros (product of costs per case and ratio of vaccinated children and RV cases avoided). Following correction for the costs for vaccination, the net cost savings per avoided case was estimated to be 19 Euros.

**Table 9:** Total costs of RV infection and cost savings (in Euros) per 1,000 children from the SHI perspective

Year	2007	2008	2009	2010	2007–2010
Overall					
Base case scenario	104,058	103,030	106,275	108,412	421,776
Inpatient	40,232	42,441	42,888	43,644	169,206
Outpatient	63,826	60,589	63,387	64,768	252,570
Vaccine scenario	102,074	101,452	91,638	86,926	382,090
Inpatient	39,880	41,804	35,609	36,183	153,476
Outpatient	62,195	59,647	56,029	50,743	228,614
Gross cost savings	1,984	1,579	14,637	21,486	39,686
Inpatient	353	637	7,279	7,461	15,730
Outpatient	1,631	942	7,358	14,025	23,956
Costs of vaccination	1,306	8,864	12,469	14,570	37,209
Net cost savings	678	−7,285	2,168	6,916	2,477
Gross cost savings per avoided case					304
Effective costs per vaccination					285
Net cost savings per avoided case					19
Cost savings due to direct and herd effects					
Gross cost savings	1,984	1,579	14,637	21,486	39,686
Herd effect	1,984	1,579	9,417	10,458	23,438
Inpatient	353	637	3,614	3,278	7,881
Outpatient	1,631	942	5,803	7,181	15,557
Direct effect	0	0	5,220	11,028	16,248

### Vaccine impact due to direct and herd effects

Table 8 shows the numbers of RV cases avoided due to herd and direct effects. The herd effect increased over time as well as the direct effect, resulting from increase in the vaccination coverage. An increasing herd effect is reasonable due to the increasing vaccination coverage, thus quantifying high external benefits of vaccination. While the unprotected age groups, i.e., children aged 3 and 4 years benefit only from the herd effect we could not quantify protection due to the herd effect for children of ages below 2 years of age. For these children, only a direct effect was observed and hence estimated. For children aged 2 and 3 years both direct and herd effects were estimated. About 59 % of the total cost savings from 2007 to 2010 was due to protection conferred as a result of the herd effect (Table 9).

### Sensitivity analysis

The effects of variation in the incidence rate (outpatient and inpatient) are summarized in Table 10. We observed an increase in total cost savings. These cost savings were disproportionately higher in inpatients than outpatients with increasing incidence rates. Herd effect was more sensitive to variations in incidence rate when compared to direct effect. In other words, to avoid a situation where the vaccine scenario exceeded the base case scenario, an increase in the incidence within the base case scenario may be an appropriate instrument. In this case, the net savings per avoided case showed the described increase. Contrarily, the net cost savings were negative when the inpatient and outpatient incidence rate was reduced by 0.06 and 0.5 %, respectively, implying that the vaccination program was not cost saving from the SHI perspective, as the costs of the vaccination program exceeded the gross savings due to implementation of the vaccination program in Saxony.

**Table 10:** Sensitivity analysis: variation of cost savings with RV inpatient and outpatient incidence rates

Variation	−0.06 %	−0.04 %	−0.02 %	Base case	+0.02 %	+0.04 %	+0.06 %
Inpatient group							
Gross cost savings (in Euros) per 1,000 children	36,909	37,802	38,736	39,686	40,646	41,660	42,674
Herd effect	21,580	22,166	22,794	23,438	24,092	24,800	25,508
Direct effect	15,330	15,636	15,942	16,248	16,554	16,860	17,166
Net cost savings per avoided case (Euros)	−2	5	12	19	26	34	41

Variation	−0.5 %	−0.4 %	−0.3 %	Base case	+0.3 %	+0.4 %	+0.5 %
Outpatient group							
Gross cost savings (in Euros) per 1,000 children	36,981	37,522	38,063	39,686	41,485	42,102	42,718
Herd effect	21,522	21,905	22,288	23,438	24,764	25,223	25,681
Direct effect	15,459	15,617	15,775	16,248	16,721	16,879	17,036
Net cost savings per avoided case (Euros)	−2	3	7	19	30	34	38

The sickness benefits represent a significant portion of the total costs and this amount depends on the employment rate of mothers, the net wage and the number of days absent from work. Although there are descriptive statistics for each of these variables, there is still incomplete information, specifically on the days absent from work. We allowed this rate to vary in a range of ±1 and ±4 days per inpatient and outpatient treatment. Table 11 shows the effect of variation in days absent from work on the total cost savings and cost savings per avoided case. Due to the relative huge proportion of sickness benefit costs on the total costs, the decrease in the number of days absent from work, especially in case of inpatient treatments, decreases the total cost savings. When reducing the days absent from work by only 1 day, the net savings are negative and implementation of vaccination is not considered to be cost saving.

**Table 11:** Sensitivity analysis: variation of cost savings with inpatient and outpatient days absent from work

Variation	−1 day	−0.5 days	Base case	+0.5 days	+1 day
Inpatient group					
Gross cost savings (in Euros) per 1,000 children	36,394	38,040	39,686	41,332	42,978
Herd effect	21,233	22,336	23,438	24,541	25,643
Direct effect	15,161	15,704	16,248	16,791	17,335
Net cost savings per avoided case (in Euros)	−6	6	19	31	44

Variation	−4 days	−2 days	Base case	+2 day	+4 days
Outpatient group					
Gross cost savings (in Euros) per 1,000 children	38,755	39,220	39,686	40,152	40,617
Herd effect	22,915	23,177	23,438	23,700	23,962
Direct effect	15,840	16,044	16,248	16,452	16,656
Net cost savings per avoided case (in Euros)	12	15	19	22	26

In Table 12 the results of reducing the vaccination costs show that the net cost savings increase up to 59 Euros when using vaccine doses from a 10-dose package.

**Table 12:** Sensitivity analysis: variation of cost savings with costs of vaccination

Variation	Base case	−5 %	−10 %	−15 %
Net cost savings per avoided case (in Euros)	19	32	46	59

## Discussion

To the best of our knowledge the presented BIA of universal RV vaccination is the first evaluation to be based on actual sick fund data. We made a distinction between age groups, type of treatment sought as well as the development of RV cases over 4 years. Most other studies have focused on the cost-effectiveness of implementing RV universal vaccination from different perspectives, including quality of life weights [32, 33].

Our analysis indicates that RV vaccination in Saxony is expected to be cost-saving from the SHI perspective. The net cost savings are expected to be 19 Euros per avoided case or 2,477 Euros per 1,000 children aged 0–5 years. Our model estimated that two children must be vaccinated to reduce the number of RV cases by one. As already indicated in previous studies [18], the herd effect indeed provided a significant contribution to cost savings due to implementation of a universal RV vaccination program in our analysis. Regarding the total cost effect, we observed that the herd effect finally dominated the direct effect. From 2007 to 2010, the herd effect increased while its relative importance decreased resulting from increasing vaccination coverage. Given the fact that the SHI regularly bears the cost of vaccination, the herd effect as an external effect is not invalidated by incentives of SHIs to reduce vaccination costs.

Previous studies in Germany have demonstrated a strong correlation between vaccination rate and a decrease in RV cases. Real-life vaccine impact data from Germany also suggest a moderate decline in RV disease burden at low and moderate levels of vaccine coverage independent of geographic location [8, 16]. It has been suggested that the reduction in incidence (population level) may have extended to children not eligible for vaccination, suggesting herd protection effects as also observed at high vaccine coverage rates in Austria [34]. Together with recently published data, our findings [8, 16] suggest that implementation of routine immunization of infants against RV could result in significant cost-savings, especially for the healthcare payer in Germany.

The sensitivity analysis showed that our assumptions relating to the days absent from work influenced the final outcomes. We expect that accounting for societal effects like saved working hours of a parent could result in higher estimates of cost savings than those calculated in this framework from the health care payer perspective. Further cost analyses, specifically on these cost components, are thus warranted.

Given the inevitable limitations of retrospective surveillance studies [35], our analysis has several limitations. First of all, the number of RV cases and the related medical costs were collected from only one specific health insurance database. Although more than half of the observed children are insured by AOK PLUS, our data might not capture the whole picture of all RV cases in Saxony. Moreover, we restricted the relevant age group to children below 5 years of age because the prevalence of RV is concentrated in this cohort. This means that the overall contribution to the herd effect by children of 5 years and older is rather neglected from the SHI perspective. The sensitivity analysis showed that our results are limited in their robustness, resulting mainly from the construction of our base case scenario, which demonstrated the development of RV cases without the opportunity of vaccination. Minimal variation in incidence, especially for inpatient cases, had a strong effect on net cost savings. The construction of the base case scenario and, due to the fact that the vaccine scenario partially exceeded the base case scenario, we may have underestimated the cost savings. Due to the distinction in inpatient and outpatient cases within the base case scenario, we assumed a constant ratio between both treatment groups taken from the actual AOK PLUS data set. However, these data are produced within the framework of the vaccination program where vaccination itself could have an influence on the inpatient and outpatient ratios. Furthermore, we may have underestimated the benefits of vaccination because the effect of the vaccination in 2010 may not have occurred until the following year, 2011, which was not included in our analysis. We also excluded any side effects of vaccination or episodes reporting vaccine failure that would potentially decrease the cost savings from the payer perspective. It is also important to note that, although in our analysis we assumed RV vaccination to be effective, which is in-line with literature, this assumption may have resulted in an overestimation of the benefits. Lastly, our cost data may have been underestimated as we did not account for children with RV infection that did not seek outpatient or inpatient care, but rather were treated at home, which is commonly the case with mild episodes of RVGE.

## Conclusions

Our retrospective analysis indicates that the impact of the recommendation for RV vaccination in Saxony has been budget neutral if not even cost saving. Economic evaluations to assess the long-term benefits of RV vaccination in Germany as well as the potential impact on prevention of hospitalization and the socioeconomic benefits of RV vaccination need to be assessed further. Prospective monitoring of RVGE cases is encouraged to obtain real-life vaccine impact data.

### Trademarks

 Rotarix is a registered trademark of GlaxoSmithKline group of companies. RotaTeq is a registered trademark of Merck and Co., United States.
